# Positive rates of total and specific immunoglobulin E in 7,824 adult patients with suspected allergic diseases in Liaoning Province, China: a retrospective study

**DOI:** 10.7717/peerj.20394

**Published:** 2025-11-21

**Authors:** Zan Sun, Long Shao, Peng Cao, Hanqi Zhang, Meng Chen, Jingfang Wang, Lin Zhou

**Affiliations:** 1 The People’s Hospital of Liaoning Province and People’s Hospital of China Medical University, Shenyang, China; 2 School of Public Health, Shenyang Medical College, Shenyang, China; 3 The First Hospital of China Medical University, Shenyang, China; 4 Liaoning University of Traditional Chinese Medicine, Shenyang, China

**Keywords:** Allergic diseases, IgE, sIgE, Positive rates

## Abstract

**Background:**

The escalating prevalence of allergic diseases poses a significant global health challenge. However, estimates of allergic disease prevalence in Liaoning Province, China, remain lacking. This study aimed to investigate total immunoglobulin E (tIgE) and specific immunoglobulin E (sIgE) levels in 7,824 patients with suspected allergic diseases and to identify factors associated with allergic conditions.

**Methods:**

A total of 7,824 participants (3,180 males and 4,644 females) with a mean age of 53.63 years were included. tIgE and sIgE levels were measured using standard laboratory methods. The normal reference range for tIgE was stratified by age group, and sIgE results were categorized as positive or negative based on predefined thresholds. Statistical analysis was performed using SPSS 26.0.

**Results:**

The overall tIgE positivity rate was 39.26%, with males showing a significantly higher rate (46.67%) than females (34.19%) (*χ*^2^ = 123.118, *p* < 0.001). The highest tIgE positivity was observed in the 18–44 age group (44.11%) and during the summer season (43.95%). No significant differences were found in sIgE positivity rates for inhaled and food allergens between sexes or seasons.

**Conclusions:**

Male sex, younger and older age groups, and the summer season were identified as significant predictors of allergic diseases based on tIgE levels. These findings underscore the importance of sex and seasonal variations in allergic disease prevalence and highlight the need for targeted prevention and management strategies.

## Introduction

Allergic diseases, characterized as chronic immune-mediated disorders, have become a global health challenge with escalating prevalence over recent decades. These conditions manifest through diverse clinical presentations including skin reactions (*e.g.*, atopic dermatitis), respiratory manifestations (*e.g.*, allergic rhinitis and asthma), gastrointestinal disturbances (*e.g.*, food allergy), and life-threatening anaphylactic shock. According to the 2011 World Allergy Organization White Book, 30%–40% of the global population suffers from at least one allergic condition (World Allergy Organization, 2011), with house dust mite (HDM) allergy affecting 65–130 million individuals worldwide (Huang, Sarzsinszky & Vrtala, 2023). A recent review confirms that food allergy is a major and growing public health concern, affecting approximately 8% of children and 10% of adults in developed countries (Bartha, Almulhem & Santos, 2024). Specifically, in the United States, food allergies affect an estimated 8% of children and 5% of adults (Gupta et al., 2018; Sicherer & Sampson, 2018). In Canada, the self-reported prevalence of food allergies increased from 7.1% to 9.3% between 2010 and 2016 (Clarke et al., 2020). Notably, in leading respiratory epidemic regions such as Australia, challenge-proven IgE-mediated food allergy now affecting up to 10% of infants (Prescott & Allen, 2011). In addition, European studies show even higher self-reported rates, with pooled lifetime and point prevalence reaching 19.9% and 13.1%, respectively (Spolidoro et al., 2023). Especially in the United Kingdom, the estimated incidence of probable food allergy doubled between 2008 and 2018, prevalence increased from 0.4% to 1.1% (Turner et al., 2024). This epidemiological surge has prompted the World Health Organization to prioritize allergy management, given its substantial socioeconomic burden through diminished quality of life and healthcare expenditures (Lai et al., 2006).

Allergic diseases arise from complex gene-environment interactions (Liu et al., 2009). The key feature of these diseases is the abnormal production of immunoglobulin E (IgE). IgE is primarily synthesized by B cells located in the lymphatic tissue of the respiratory and digestive tracts’ lamina propria mucosa. As the primary mediator of type I allergic reactions, IgE plays a central role in these conditions. Elevated serum IgE levels often suggest the presence of genetic allergies or type I hypersensitivity. Though the underlying mechanisms are not fully elucidated, emerging evidence highlights demographic influences on disease manifestation, with age-related susceptibility patterns (Tuten Dal et al., 2024), sex disparities in immune responses (Osman, 2003), and seasonal variations in allergen exposure (Lee et al., 2020) being increasingly recognized. These findings underscore the necessity for multidimensional approaches in clinical management, emphasizing personalized prevention and treatment strategies.

Despite global research advances, significant knowledge gaps persist regarding regional epidemiological variations. Liaoning Province, a northeastern Chinese region characterized by distinct seasonal transitions with prolonged heating periods and associated indoor allergen exposure, likely presents unique allergy profiles. However, the absence of comprehensive epidemiological data from this 43-million population region hinders targeted interventions. This study aimed to investigate the total and specific IgE levels in 7,824 adult patients with suspected allergic diseases and identify the factors associated with allergic diseases. The findings from this investigation are expected to provide valuable insights for the development of more effective diagnostic tools, preventive strategies, and therapeutic interventions tailored to the needs of individuals at risk for allergic diseases.

## Methods

### Study population

In this retrospective study, the results of testing 7,824 patients with suspected allergies who attended the Liaoning Provincial People’s Hospital in China between January 2018 and December 2023 were analyzed. These patients were clinically evaluated by the attending physicians and all presented with suspected allergic symptoms, including atopic dermatitis, urticaria, eczema, asthma, shortness of breath, cough, and respiratory tract infections. The investigation was reviewed and approved by the Ethics Committee of The People’s Hospital of Liaoning Province (2024K004). Since this study was a retrospective study form, the application for waiver of informed consent was adopted.

### Allergy screen test

#### Preparation of blood sample and reagents

The collected blood was placed in a sterile drying tube or a coagulant tube containing separation gel. The sample should be centrifuged immediately upon receipt at 3,000 rpm for 10 min to facilitate serum separation. If immediate testing was not feasible, serum samples must be stored under cold conditions at 2–8 °C and tested as soon as possible. Samples exhibiting severe hemolysis or lipemia were deemed unsuitable for this experiment due to their potential interference with result analysis.

Antibody levels in the serum of patients were evaluated using the Total IgE Antibody Detection Kit (Catalog No.: MB00072), the Allergen-Specific IgE Antibody (sIgE) Detection Kit for Inhaled Allergens (Catalog No.: MB00061), and the sIgE Detection Kit for Food Allergens (Catalog No.: MB00062), all manufactured by HOB Biotech Group Corp., Ltd. (Suzhou, Jiangsu, China). Experiments were performed in strict accordance with the instructions provided. The reagents in the kits were equilibrated for 30 min at room temperature, and the working concentration of the wash solution was obtained by diluting the concentrated wash solution with purified or distilled water at a ratio of 1:10. The prepared wash solution should be used as soon as possible, and the unused portion must be stored at a low temperature of 2–8 °C.

#### Test procedure

The incubators with the test strips were placed on a shaker, and one mL of serum sample was added to each incubator followed by incubation with shaking at 40 rpm for 60 min at room temperature. After discarding the reaction solution in the incubator, the test strips were washed with the prepared washing solution and the above washing procedure was repeated three times. Then one mL of bonding solution was added to each test strip and incubated for 20 min at room temperature with shaking at 40 rpm, followed by two washes with washing solution. one mL of substrate solution was then added to each incubator and incubation continued for 10 min at room temperature. Finally, the test strips were removed, and excess liquid was blotted with absorbent paper and left to dry completely before placing it on the reading system for interpretation of results.

The obtained test strips were analyzed by a Rayto automated immunoblotting instrument (Shenzhen, China) to achieve qualitative classification of allergen-specific IgE. Each strip contains a negative and a positive control area. The color of the negative control area appeared white or extremely light blue, while the color of the positive control area was significantly more intense than that of the negative control zone, indicating a valid result. If the color intensity of the allergen response zone exceeded that of the negative control zone, it was interpreted as a positive reaction, signifying the presence of specific IgE for that allergen in the patient’s serum. In contrast, if the antigen reactive area was the same or similar in color to the negative control area, a negative reaction was indicated, suggesting that IgE specific for the allergen was not present in the patient’s serum. It is crucial to emphasize that chromogenic results from total IgE response regions do not serve as substitutes for quantification *via* enzyme-linked immunosorbent assay (ELISA) methods recommended by the World Health Organization (WHO), and these results are only intended to predict IgE-mediated allergic reactions.

#### Evaluation of tIgE and sIgE

The normal reference range of tIgE can be categorized as follows: 3 to 5 years old, tIgE range was 0 to 35 IU/mL; 6 to 20 years old, tIgE range was 0 to 51 IU/mL; over 20 years old, tIgE range was 0 to 100 IU/mL. Patients with tIgE values outside the reference range were considered positive. sIgE test results <0.35 IU/mL were negative and ≥ 0.35 IU/mL were positive. A case was considered positive whenever 1 or more sIgE were positive in the patient’s serum.

### Statistical analysis

SPSS 26.0 software (IBM Corp., Armonk, NY, USA) was used for statistical analysis of the data, and comparisons between groups were performed by Chi-square test. *P* <0.05 was considered to be statistically significant.

## Results

### Characteristics of the study population

**Figure 1:**
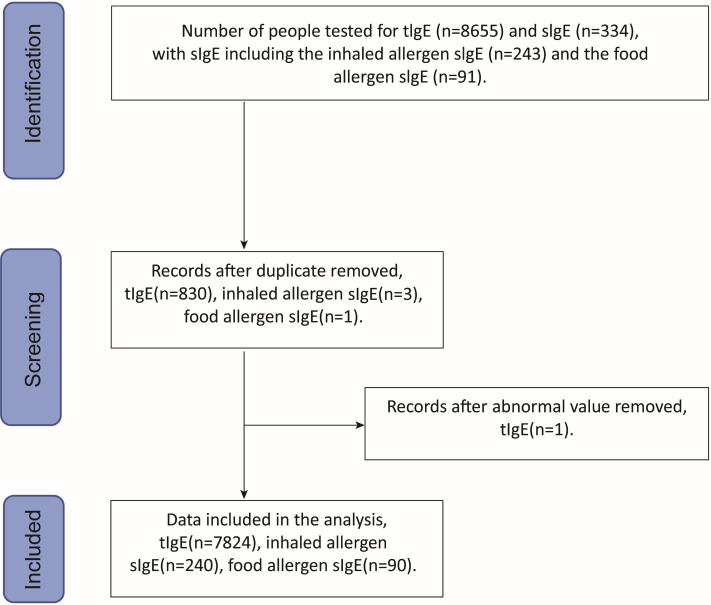
Data screening flowchart.

**Figure 2:**
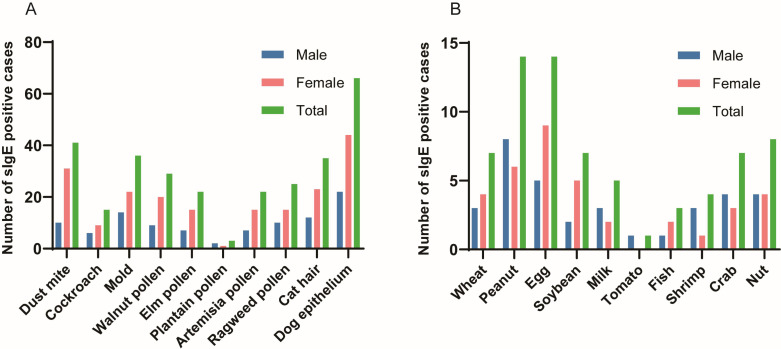
Positive rates for 10 inhaled allergens and 10 food allergens. (A) Inhaled allergens; (B) Food allergens.

Of the 7,824 patients with suspected allergies, the number and percentage of patients tested for tIgE and sIgE varied by year as follows: 823 (10.52%) in 2018, 1,157 (14.79%) in 2019, 762 (9.74%) in 2020, 1,334 (17.05%) in 2021, 1,639 (20.95%) in 2022, and 2,109 (26.95%) in 2023. As shown in Fig. 1, the subjects comprised 3,180 males (40.64%) and 4,644 females (59.36%), with a mean age of 53.63 ± 18.32 years (range: 18 years to 101 years). Among them, 7,824 patients underwent total IgE detection and 330 patients were tested for specific IgE detection, including 240 cases with 10 inhaled allergen-specific IgE tests and 90 cases with 10 food allergen-specific IgE tests. As shown in Fig. 2, allergens tested covered 20 common allergens, 10 inhaled allergens and 10 food allergens. Inhaled allergens include dust mite, cockroach, mold, walnut pollen, elm pollen, plantain pollen, artemisia pollen, ragweed pollen, cat hair, dog epithelium, while food allergens included wheat, peanut, egg, soybean, milk, tomato, fish, shrimp, crab, and nuts.

### Analysis of tIgE and sIgE by sex

#### Overall analysis of tIgE and sIgE by sex

It can be seen from Table 1, out of 7,824 patients with total IgE, 3,180 were male, of whom 1,484 were positive for total IgE. Out of 4,644 female patients, 1,588 were positive for total IgE. The total positive rate of total IgE in serum was 39.26%, with 46.67% in male patients and 34.19% in female patients. The difference in total IgE positivity between the male and female groups was statistically significant (*χ*2 = 123.118, *P* < 0.001). Among the sIgE-positive cases for the 10 inhaled allergens, 43 males and 87 females were positive, with the difference also not being statistically significant (*χ*2 = 0.15, *P* > 0.05) whereas among the sIgE-positive cases for the 10 food allergens, 14 cases were male and 18 cases were female, with no statistical significance (*χ*2 = 0.01, *P* > 0.05).

**Table 1:** Positive rates of tIgE and sIgE.

Gender	tIgE (N)		Inhaled allergens (N)		Food allergens (N)	
	n	tIgE(+)(%)	n	sIgE(+)(%)	n	sIgE(+)(%)
Male	3,180	1,484 (46.67)	82	43 (52.44)	40	14 (35.00)
Female	4,644	1,588 (34.19)	158	87 (55.06)	50	18 (36.00)
*χ2*		123.118		0.15		0.01
*P*		< 0.001		0.699		0.922

#### Sensitization to inhaled and food allergens by sex

As shown in Table 2, out of 240 patients, 130 (54.17%) tested positive for sIgE to at least one of the 10 inhaled allergens, with dog epithelium (66, 27.50%), dust mites (41, 17.08%), and molds (36, 15.00%) being the most common inhaled allergens. Out of 90 patients, 32 (35.56%) tested positive for sIgE to at least one of the 10 food allergens, with peanuts (14, 15.56%), eggs (14, 15.56%), and nuts (8, 8.89%) being the top three food allergens.

#### Analysis of inhaled allergens and food allergens in different sex groups

Table 3 show the sex distribution of 10 inhaled allergens and 10 food allergens. As shown in Table 3 and Fig. 2, the top inhaled allergen was dog epithelium in both males and females, with positivity rates of 26.83% and 27.85%, respectively. In addition, mold (17.07%) and dust mite (19.62%) were the second most common inhaled allergens in male and female patients, respectively (Table 3 and Fig. 2). It can be seen from Table 3 and Fig. 2 that the food allergens in the patients were mainly eggs and peanuts, the food allergens in the male patients were mainly peanuts, and the food allergens in the female patients were mainly eggs. Peanut (20%) and egg (12.5%) were the common food allergens in male patients, whereas egg (18%), peanut (12%) and soybean (10%) were the most common food allergens in female patients However, due to the lack of sample size, there was no statistical significance in the sex distribution of specific IgE.

**Table 2:** Sensitization to 10 inhaled and 10 food allergens.

Inhaled allergens	Positive number (%)	Food allergens	Positive number (%)
Dust mite	41 (17.08)	Wheat	7 (7.78)
Cockroach	15 (6.25)	Peanut	14 (15.56)
Mold	36 (15.00)	Egg	14 (15.56)
Walnut pollen	29 (12.08)	Soybean	7 (7.78)
Elm pollen	22 (9.17)	Milk	5 (5.56)
Plantain pollen	3 (1.25)	Tomato	1 (1.11)
Artemisia pollen	22 (9.17)	Fish	3 (3.33)
Ragweed pollen	25 (10.42)	Shrimp	4 (4.44)
Cat hair	35 (14.58)	Crab	7 (7.78)
Dog epithelium	66 (27.50)	Nut	8 (8.89)

**Table 3:** Sensitization to 10 inhaled and 10 food allergens by gender.

Inhaled allergens	Male (*n* = 82)	Female (*n* = 158)	*χ*2	*P*	Food allergens	Male (*n* = 40)	Female (*n* = 50)	*χ*2	*P*
	Positive rate (%)	Positive rate (%)				Positive rate (%)	Positive rate (%)		
Dust mite	10(12.20)	31(19.62)	2.101	0.147	*Wheat* ^a^	3(7.50)	4(8.00)	0	1
Cockroach	6(7.32)	9(5.70)	0.242	0.623	Peanut	8(20.00)	6(12.00)	1.083	0.298
Mold	14(17.07)	22(13.92)	0.420	0.517	Egg	5(12.50)	9(18.00)	0.512	0.474
Walnut pollen	9(10.98)	20(12.66)	0. 144	0.704	*Soybean*	2(5.00)	5(10.00)	0.234	0.628
Elm pollen	7(8.54)	15(9.49)	0.059	0.807	*Milk*	3(7.50)	2(4.00)	0.066	0.797
*Plantain pollen* ^a^	2(2.44)	1(0.63)	0.339	0.561	**Tomato** ^b^	1(2.50)	0(0.00)		0.444
Artemisia pollen	7(8.54)	15(9.49)	0.059	0.807	*Fish*	1(2.50)	2(4.00)	0	1
Ragweed pollen	10(12.20)	15(9.49)	0.422	0.516	*Shrimp*	3(7.50)	1(2.00)	0.553	0.457
Cat hair	12(14.63)	23(14.56)	0.000	0.987	*Crab*	4(10.00)	3(6.00)	0.095	0.758
Dog epithelium	22(26.83)	44(27.85)	0.028	0.867	*Nut*	4(10.00)	4(8.00)	0	1

**Notes.**

a Italic: correction formula.

b Bold: Fisher’s exact test.

### Positive rates of tIgE by age

As shown in Table 4, the adult patients investigated were categorized into three groups, 18–44 years, 45–59 years and 60 years and above according to the World Health Organization criteria. The highest positive rate was found in the age group of 18–44 years (44.11%), followed by the age group of ≥60 years (37.45%) and 45–59 years (35.61%). The difference was statistically significant (*χ*2 = 41.096, *P* < 0.001).

**Table 4:** Positive rates of tIgE by age.

Age (years)	tIgE (N)	
	n	tIgE (+)(%)
18–44	2,648	1,168 (44.11)
45–59	1,873	667 (35.61)
60–101	3,303	1,237 (37.45)
*χ*2		41.096
*P*		<0.001

### Positive rates of tIgE and sIgE by season

According to international meteorological standards, the year is divided into four seasons, of which spring is from March to May, summer is from June to August, autumn is from September to November, and winter is from December to February. As can be seen in Table 5, the positive rate of tIgE showed that the highest positivity rate was observed in summer (43.95%), followed by autumn (40.19%), spring (37.21%) and winter (34.06%). tIgE positive rate displayed a statistically significant difference among the seasons (*χ*2 = 42.127, *P* < 0.001). The number of sIgE positive cases for the 10 inhaled allergens in spring, summer, autumn, and winter were 30, 40, 35, and 25, respectively, with no statistically significant difference (*χ2* = 5.492, *P* > 0.05). Similarly, the number of sIgE positive cases for the 10 food allergens in spring, summer, autumn, and winter were 7, 7, 9, and 9, respectively, with no statistically significant difference (*χ2* = 1.085, *P* > 0.05).

**Table 5:** Positive rates of tIgE and sIgE by seasons.

Season	tIgE (N)		Inhaled allergens (N)		Food allergens (N)	
	n	tIgE (+)(%)	n	sIgE (+)(%)	n	sIgE (+)(%)
Spring	2,021	752 (37.21)	70	30 (42.86)	20	7 (35.00)
Summer	2,198	966 (43.95)	71	40 (56.34)	21	7 (33.33)
Autumn	2,055	826 (40.19)	59	35 (59.32)	29	9 (31.03)
Winter	1,550	528 (34.06)	40	25 (62.50)	20	9 (45.00)
*χ*2		42.127		5.492		1.085
*P*		<0.001		0.139		0.781

### Clinical symptoms and tIgE positivity among patients with suspected allergy

The ten most common clinical symptoms and tIgE positive rates among the 7,824 patients are shown in Table 6, which indicated that there were significant differences in tIgE positivity among different clinical conditions. Among them, urticaria, dermatitis and rhinitis had the highest tIgE positivity rates of 66.86%, 62.54% and 45.38%, respectively, suggesting that these symptoms were more closely related to allergic reactions. Overall, these results contribute to a clinician’s better understanding of the relationship between different symptoms and immune system responses when diagnosing and treating allergic diseases.

**Table 6:** Top ten most common clinical symptoms and tIgE positivity among patients with suspected allergy.

Clinical symptoms	Frequency (n)	tIgE (+) (%)
Pneumonia	2,306	391 (16.96)
Hypertension	1,368	194 (14.18)
Asthma	1,107	258 (23.31)
Dermatitis	1,009	631 (62.54)
Chronic Obstructive Pulmonary Disease	757	118 (15.59)
Urticaria	679	454 (66.86)
Bronchitis	654	202 (30.89)
Respiratory Failure	638	99 (15.52)
Type 2 Diabetes	620	107 (17.26)
Rhinitis	368	167 (45.38)

## Discussion

In this study, the levels of total IgE and specific IgE were systematically analyzed in 7,824 adult patients with suspected allergies. In agreement with some previous studies (Ballardini et al., 2021; Park et al., 2017), it is observed that tIgE positivity is significantly higher in male patients than in female patients. This may be due to hormonal differences as estrogen is known to modulate the immune response and potentially prevent sensitization (Bonds & Midoro-Horiuti, 2013). However, other studies have also reported that sex differences are not significant and even higher prevalence in females, suggesting that the relationship between sexes and allergic diseases is complex and may be influenced by a variety of factors such as genetics, individual lifestyle habits and environmental exposure (Lokaj-Berisha et al., 2015; Rosário et al., 2021; Tagliaferro et al., 2024). Genome-wide association studies (GWAS) have identified genetic risk loci for asthma and allergy that show sex-specific effects, underscoring the intricate interplay between genetics and sex in allergic inflammation (Moffatt et al., 2010; Verlaan et al., 2009). Beyond genetic predisposition, behavioral differences may also play a role; for example, women tend to pay more attention to skin care and the use of cosmetics, which contain some chemicals and fragrances that may become potential allergens and increase the chance of allergic diseases in women. In contrast, men may have different exposures to other specific allergens due to factors such as their occupations, which may lead to the difference in the incidence of allergic diseases between the sexes (De Martinis et al., 2020). Furthermore, a previous study has suggests that high alcohol consumption is associated with elevated tIgE levels in men (Carballo et al., 2021).

This study reveals higher tIgE positivity in younger and older adults. Although the finding in younger individuals is similar to previous reports (Omenaas et al., 1994), the result in the older group contrasts sharply with earlier findings (Viswanathan & Mathur, 2011). It is related to the different lifestyles and exposure to the environment in different age groups, and thus can influence the development of allergic diseases (Fang et al., 2023). For example, younger people may be more exposed to outdoor allergens. Furthermore, previous studies have also reported that in healthy individuals, the total serum IgE level gradually increases from birth to the age of 15, and then gradually decreases from the age of 20 to 80 (Barbee et al., 1981). However, the elderly exhibit increased susceptibility to infections, autoimmune diseases, and malignancy due to the functional decline of both innate and adaptive immunity (Derhovanessian et al., 2008), which makes them more susceptible to allergic reactions (Huang et al., 2022; Kousa et al., 2024; Sadighi Akha, 2018), resulting in a higher tIgE positivity rate.

Furthermore, we have observed that the tIgE positivity rate of the investigated group of adult suspected allergy sufferers is the highest in summer, followed by autumn. This pattern contrasts with the established pollen seasons in Liaoning Province, where spring (typically late March to mid-June) is dominated by poplar and autumn (typically August to September) by ragweed pollen (Zhang et al., 2024). This seasonal distribution is partially supported by a Canadian study showing that the number of positive Radioallergosorbent Test (RAST) results peaked in April and November (Salkie & Weimer, 1984). This divergence suggests that while pollen exposure contributes to allergic sensitization, the elevated tIgE levels observed in summer may also be partially attributable to parasitic co-infections. National surveys indicate that while soil-transmitted helminth infections are most prevalent in southwestern provinces like Yunnan (11.83%) and Hainan (10.9%) (Chen et al., 2021; Zhu et al., 2019), certain foodborne parasitic diseases show significant prevalence in northeastern China. Specifically, *Clonorchis sinensis* (Chinese liver fluke) infection is highly endemic in Heilongjiang and Jilin provinces, with these regions identified as major endemic areas alongside Guangdong and Guangxi. Although data specific to Liaoning Province have not been explicitly reported, populations in this region may still face similar exposure risks due to its geographical proximity and comparable environmental conditions. The warm, humid summer environment not only promotes the transmission of these parasites but may also enhance their immunogenicity, thereby amplifying IgE responses (Pullan & Brooker, 2008). Additionally, summer conditions facilitate the proliferation of other allergens such as fungi or dust mites, as well as elevated temperature and air humidity. These environmental factors not only promote the multiplication of common allergens but also may increase people’s chances of coming into contact with these allergens, thus exacerbating allergic symptoms. The tIgE positivity rate in this study population is lower in the winter months. This seasonal pattern aligns with findings from a previous dynamic study by Korean scholars, which reported a significant reduction in both total serum IgE and specific IgE antibody levels from September to December (Nahm et al., 1997). The reason for the above result might be that in winter, the body may dedicate more immune resources to responding to cold-weather threats of infections such as influenza viruses, respiratory syncytial viruses, *etc*. (Zuo et al., 2021). During this state of immune stress, the number and function of regulatory T cells (Tregs) are enhanced to maintain immune homeostasis and suppress excessive immune responses, including allergic reactions mediated by helper T cells 2 (Th2) (Robinson, Larché & Durham, 2004). Th2 cells are important regulators of IgE production, and inhibition of their activity leads to a decrease in the level of IgE produced by B cells, resulting in lower total IgE levels observed in winter (Deo et al., 2010; Goetzl, 2024).

In addition, the suspected allergic patients in this study involved a variety of clinical conditions, such as urticaria, dermatitis, rhinitis, and asthma. There is an intrinsic link between different types of allergic clinical symptoms with similar pathogenesis or common risk factors. For example, atopic dermatitis is often considered to be an early manifestation of allergic disease development, and patients are at higher risk of developing allergic rhinitis and asthma later in life (Mrkić Kobal et al., 2023; Yang, Fu & Zhou, 2020). It has to be mentioned that many patients with suspected allergies suffer from other chronic diseases such as cardiovascular disease and metabolic syndrome. Studies have shown that patients with allergic diseases may have an increased risk of developing cardiovascular disease, which may be related to inflammatory responses, immune imbalances and other mechanisms (Guo et al., 2022; Paller et al., 2018; Silverberg et al., 2018). In this study, some of the patients had co-morbidities such as hypertension and diabetes, which can affect the risk, severity and IgE levels of allergic diseases. For example, patients with hypertension may be on certain medications for long periods of time that affect the immune system, which can alter sensitivity to allergens (Kostis et al., 2018; Lieberman & Simons, 2015). Patients with diabetes may have exacerbated allergies due to poor glycemic control and chronic inflammation (Lei et al., 2024). In conclusion, studying the associations between these different types of allergic diseases and with other diseases can help in the early identification of at-risk populations, leading to more targeted prevention and intervention.

Emerging evidence now demonstrates that, in addition to traditional mechanisms, a complex interplay between immune pathways and environmental modulators is crucial. Th17 cells exhibit dual roles in IgE regulation, not only promoting inflammation *via* IL-17 but also potentiating Th2 responses (Cosmi et al., 2010; Murdaca, Colombo & Puppo, 2011). This crosstalk suggests Th17 cells as potential therapeutic targets for allergic diseases. The IL-31/IL-33 axis further complicates the Th2-dominant paradigm. IL-33, as an “alarmin,” triggers IL-31 release from Th2 cells (Di Salvo et al., 2018; Murdaca et al., 2019), while dysregulation of this axis exacerbates IgE-mediated allergies (Murdaca, Gangemi & Greco, 2023; Murdaca et al., 2019). Such findings underscore the need for cytokine-network-based therapies. Environmental-metabolic interactions also contribute to IgE dysregulation. Vitamin D deficiency disrupts gut microbiota homeostasis, thereby impairing immune balance (Murdaca et al., 2021). Furthermore, research indicates that vitamin D deficiency may lead to an increased incidence of allergies and asthma (Mirzakhani et al., 2015; Sikorska-Szaflik & Sozańska, 2020). Notably, vitamin D supplementation can reshape microbial composition and modulate immune cells *via* vitamin D receptors (Murdaca & Gangemi, 2022; Murdaca & Gangemi, 2024; Yamamoto & Jørgensen, 2019), highlighting an epigenetic link between environment and immunity. Furthermore, immune tolerance mechanisms may counterbalance IgE overproduction. Elevated sHLA-G in allergic patients (Murdaca, Colombo & Puppo, 2011; Negrini et al., 2021) implies compensatory tolerance activation, offering insights into endogenous regulatory checkpoints. Together, these mechanisms converge into a multi-layered IgE regulatory network, advancing our understanding of allergic pathogenesis and informing targeted interventions.

While this study provides valuable insights into the epidemiology of allergic diseases and emphasizes the importance of considering sex, age and seasonal factors in the development and management of allergic diseases. However, this study still has several limitations that should be considered when interpreting the results. First, the cross-sectional design of the study limits our ability to establish a causal relationship between the factors analyzed and the incidence of allergic diseases. Second, due to potential selection bias, the study population may not be fully representative of the general population, and the results of this study are only applicable to Liaoning, China, or areas with similar climatic conditions, species richness, and lifestyles. Notably, due to the lack of sIgE data, the current analysis may not be able to fully reflect the patterns of responses to specific allergens in different populations, nor can it provide insight into the exposure to specific allergens and their effects on disease occurrence in different seasons or age groups. Third, this study incorporated 7,824 independent, non-repeated samples. Due to the inherent limitations of the data, a longitudinal analysis of seasonal variations in tIgE levels within the same patients was not feasible. Consequently, these data preclude drawing definitive conclusions regarding the precise effect of season on tIgE. Finally, when discussing the relationship between clinical symptoms and tIgE and sIgE positive rates, the present study lacked specific data to support the relationship, although it mentioned that there might be an interaction. The lack of detailed collection of specific information about the patients’ conditions, such as the severity and duration of the disease, limited the in-depth understanding of the relationship. It is worth noting that, apart from allergies, total IgE levels are also influenced by a variety of other conditions, such as parasitic infections (Belhassen-García et al., 2014; Hamid et al., 2015; Yasuda & Nakanishi, 2018), immunodeficiencies (Boos et al., 2014; Tsilifis, Freeman & Gennery, 2021), and autoimmune diseases (De Oliveira et al., 2015; Kolkhir et al., 2017). Therefore, an elevated serum IgE level does not equate to an allergy diagnosis. In summary, future studies should increase the sample size of sIgE testing in order to more comprehensively assess the risk factors and epidemiologic characteristics of allergic diseases. In addition, to better understand the relationship between allergic diseases and potential risk factors, future studies should utilize longitudinal designs and include larger and more diverse study populations.

## Conclusions

This study investigated the tIgE and sIgE positivity rates of 7,824 patients with suspected allergies. The higher tIgE positivity rate in male patients compared to female patients suggests that male patients are more susceptible to allergic diseases. In addition, younger and older age groups also had higher tIgE positivity rates, implying that these age groups are more prone to allergic diseases. Further, tIgE positivity rates were highest during the summer season, suggesting that allergic diseases are more likely to occur during the summer season. Although this study provides important information on the relationship between tIgE positivity and allergic diseases, it is worth noting that the number of people tested for sIgE was small, which limits more in-depth t and precise analysis. These findings could provide important guidance for clinical practice, as well as a basis for further research into the pathogenesis of allergic diseases and the development of personalized preventive measures.

##  Supplemental Information

10.7717/peerj.20394/supp-1 Supplemental Information 1 Raw data Time of visit, gender, age, diagnosis, sIgE and tIgE results for all patients.

10.7717/peerj.20394/supp-2 Supplemental Information 2 STROBE checklist

## Additional Information and Declarations

### Data Availability

The following information was supplied regarding data availability: The raw measurements are available in the Supplementary File.
