# Supplementary material for: Positive rates of total and specific immunoglobulin E in 7,824 adult patients with suspected allergic diseases in Liaoning Province, China: a retrospective study
Source: PeerJ. 2025 Nov 21;13:e20394. doi: 10.7717/peerj.20394 (PMC12642912; doi:10.7717/peerj.20394)
Supplement: Supplemental Information 2 [file peerj-13-20394-s002.doc]

STROBE Statement—checklist of items that should be included in reports of observational studies

|  | Item No | Recommendation |
| --- | --- | --- |
| **Title and abstract** | 1 | (*a*) Title ("Positive rates of total and specific Immunoglobulin E in 7824 adult patients with suspected allergic diseases in Liaoning Province, China") |
|  |  | (*b*) Abstract (Background, Methods, Results, Conclusions sections) |
| Introduction | | |
| Background/rationale | 2 | Introduction (Global prevalence of allergies, regional gaps in Liaoning, IgE's role in allergies) |
| Objectives | 3 | Abstract ("This study aimed to investigate...") and Introduction (final paragraph) |
| Methods | | |
| Study design | 4 | Methods 2.1 ("retrospective study"), Abstract ("cross-sectional analysis") |
| Setting | 5 | Methods 2.1 (Liaoning Provincial People’s Hospital, January 2018–December 2023) |
| Participants | 6 | (*a*) Methods 2.1 (patients with suspected allergic symptoms) |
|  |  | (*b*) N/A (No matching was performed) |
| Variables | 7 | Methods 2.2.3 (tIgE/sIgE thresholds), Results 3.1 (variables: age, gender, season) |
| Data sources/ measurement | 8* | Methods 2.2.1–2.2.2 (blood sample processing, test kits, interpretation) |
| Bias | 9 | Limitations mention potential selection bias but no specific mitigation steps. |
| Study size | 10 | No explanation for sample size (7,824 participants). |
| Quantitative variables | 11 | Methods 2.2.3 (tIgE/sIgE categorization), Results 3.3 (age groups) |
| Statistical methods | 12 | (*a*) Methods 2.4 (Chi-squared tests, SPSS 26.0) |
|  |  | (*b*) Results 3.2–3.4 (gender, age, season subgroups) |
|  |  | (*c*) Not mentioned. |
|  |  | (*d*) N/A (Cross-sectional design) |
|  |  | (*e*) Not performed. |
| Results | | |
| Participants | 13* | (a) Results 3.1 (7,824 participants, breakdown by year/gender) |
|  |  | (b) Not provided. |
|  |  | (c) Figure 1 |
| Descriptive data | 14* | (a) Results 3.1 (demographics), Table 1 (gender distribution) |
|  |  | (b) Not reported. |
|  |  | (c) N/A |
| Outcome data | 15* | Results 3.2–3.5 (positive rates by gender/age/season) |
| Main results | 16 | (*a*) Unadjusted estimates (Chi-squared tests) are provided. |
|  |  | (*b*) Methods 2.2.3 (tIgE/sIgE thresholds) |
|  |  | (*c*) N/A |
| Other analyses | 17 | Results 3.2–3.4 (subgroups by gender/age/season) |
| Discussion | | |
| Key results | 18 | Discussion (first paragraph) |
| Limitations | 19 | Discussion (cross-sectional design, selection bias, sIgE sample size) |
| Interpretation | 20 | Discussion (contextualized with prior studies, hormonal/environmental factors) |
| Generalisability | 21 | Discussion (regional applicability, limitations) |
| Other information | | |
| Funding | 22 | Funding (Natural Science Foundation of Liaoning Province), Declarations |

*Give information separately for cases and controls in case-control studies and, if applicable, for exposed and unexposed groups in cohort and cross-sectional studies.

**Note:** An Explanation and Elaboration article discusses each checklist item and gives methodological background and published examples of transparent reporting. The STROBE checklist is best used in conjunction with this article (freely available on the Web sites of PLoS Medicine at http://www.plosmedicine.org/, Annals of Internal Medicine at http://www.annals.org/, and Epidemiology at http://www.epidem.com/). Information on the STROBE Initiative is available at www.strobe-statement.org.
